# Multifunctional Cellulosic Natural Rubber and Silver Nanoparticle Films with Superior Chemical Resistance and Antibacterial Properties

**DOI:** 10.3390/nano13030521

**Published:** 2023-01-28

**Authors:** Goragot Supanakorn, Siriporn Taokaew, Muenduen Phisalaphong

**Affiliations:** 1 Bio-Circular-Green-Economy Technology & Engineering Center, BCGeTEC, Department of Chemical Engineering, Faculty of Engineering, Chulalongkorn University, Bangkok 10330, Thailand; 2 Department of Materials Science and Biotechnology, School of Engineering, Nagaoka University of Technology, Nagaoka 940-2188, Japan

**Keywords:** silver nanoparticle, natural rubber, cellulose fiber, biocomposite film

## Abstract

Composite films of natural rubber/cellulose fiber/silver nanoparticle were synthesized in a green route via the latex solution process. Hybrid cellulose filler containing carboxymethyl cellulose and cellulose microfibers was used to facilitate facile and fast preparation and to improve mechanical strength to the composites, respectively. All the composites possessed a high tensile strength of ~120 MPa, a high heat resistance of nearly 300 °C, and more than 20% biodegradability in soil in two weeks. Chemical resistance and antibacterial activity of the composite was enhanced depending on sizes and concentrations of silver nanoparticles (AgNPs). The composites containing 0.033–0.1% *w*/*w* AgNPs retarded toluene uptake to less than 12% throughout 8 h, whereas the composite containing 0.067–0.1% *w*/*w* AgNPs exhibited excellent antibacterial activities against *Escherichia coli* and *Staphylococcus aureus*. In comparison, 50 nm-AgNPs presented higher antibacterial activities than 100 nm-AgNPs. In vitro cytotoxicity test assessed after incubation for 24 h and 48 h revealed that almost all AgNPs-composite films exhibited non/weak and moderate cytotoxicity, respectively, to HaCaT keratinocyte cells.

## 1. Introduction

The utilization of green processes for the development of biopolymer composites plays an important role in minimizing environmental impacts [1]. Green preparation using facile routes has many potential advantages in producing new materials with reduced waste and lower energy consumption [2]. In contrast, as considered in the life cycle assessment, complex methods tend to be more harmful to environments due to increased energy consumption and waste generation [3,4].

Natural rubber (NR) is the biopolymer cis-1,4-polyisopene comprised of isoprene as a monomer containing a double bond in the cis configuration. Natural rubber latex (NRL) is a natural product from rubber trees (*Hevea brasiliensis*). NRL is widely used in various elastic products [5,6]; however, pure natural rubber products have relatively low mechanical strength and organic solvent resistance, with no antimicrobial properties [7,8]. Therefore, natural rubber is usually chemically modified by the vulcanization process to improve the mechanical properties of neat rubber. The linear chains of the rubber are cross-linked with covalent bonds and consequently the vulcanized molecular network is strengthened [9,10]. However, the main drawbacks of this process are waste generation and energy consumption [9,10]. Rubber modification via the addition of natural fillers has been employed to overcome and eliminate the use of hazardous chemicals and harsh condition in the preparation and modification of the rubber [11]. Hybrid filler reinforcement is a fascinating approach to combine the properties of the fillers in the composite. For instance, cellulose fiber and sodium carboxymethyl cellulose (SCMC) is a particularly interesting filler pairing since the NR composited with this combination of fillers exhibits outstanding mechanical properties, chemical resistance, and biodegradability [12]. Cellulose is a polysaccharide consisting of a linear chain of β-1,4 linked D-glucose units. The cellulose fibrils, having an abundance of hydroxyl groups, are formed through inter- and intramolecular hydrogen bonding, leading to high crystallinity [13]. Hence, it has been used as a green filler to achieve pronounced improvement of mechanical properties. To improve compatibility between the hydrophobic NR and the hydrophilic cellulose, the secondary amphiphilic additive, such as sodium alginate and sodium carboxymethyl cellulose is required [11,12]. Sodium carboxymethyl cellulose (SCMC), a linear β-1,4 linked D- glucopyranose polymer, is an anionic water-soluble cellulose derivative widely used as a stabilizer in food [14]. With amphiphilic characteristics from the combination of a hydrophobic backbone and many carboxyl groups [15], SCMC lowers the polarity difference between NR and cellulose to reduce the agglomeration of cellulose fibers and subsequently shortens the blending time to preserve the unstable nature of NRL [16]. Besides acting as a pre-agglomeration stabilizer to obtain high dispersion of the primary cellulose filler in NRL, SCMC is also a stabilizer of silver nanoparticles (AgNPs) [17], which is adopted to enhance antimicrobial property to NR composites in this study. AgNP-based biopolymers offer good biocompatibility and biodegradability and can be considered as an effective material for application in antimicrobial packaging and pharmaceutical and medical fields [18,19,20,21]. The antimicrobial effect of Ag has been previously demonstrated against many types of microorganisms, including bacteria, viruses, and protozoa [18,19,20,21]. It has been shown that AgNPs exhibit strong antibacterial activities against both Gram-positive and Gram-negative bacteria. The complex antimicrobial mechanism of AgNPs involves multiple targets, including damaging the bacterial cell membrane, disruption of DNA replication, and eventually apoptosis [22]. Hence, they have been extensively applied as an antibacterial agent in biomedical applications and food packaging [23,24]. However, their antimicrobial effect varies with size, shape, and synthesis method, i.e., chemical and biological techniques [25,26].

This research aims to integrate the advantages of hybrid fillers to obtain rubber composites with multifunctional properties, especially antimicrobial properties. A combination of cellulose microfibers (CF), SCMC, and AgNPs were adopted as hybrid filler. Based on the green preparation concept, NR composite films were prepared by mechanically blending with the fillers via a latex aqueous microdispersion process. The effects of particle sizes and concentrations of AgNPs on the physical, chemical, and biological properties of NR composite films were investigated.

## 2. Materials and Methods

### 2.1. Materials

**Figure 1:**
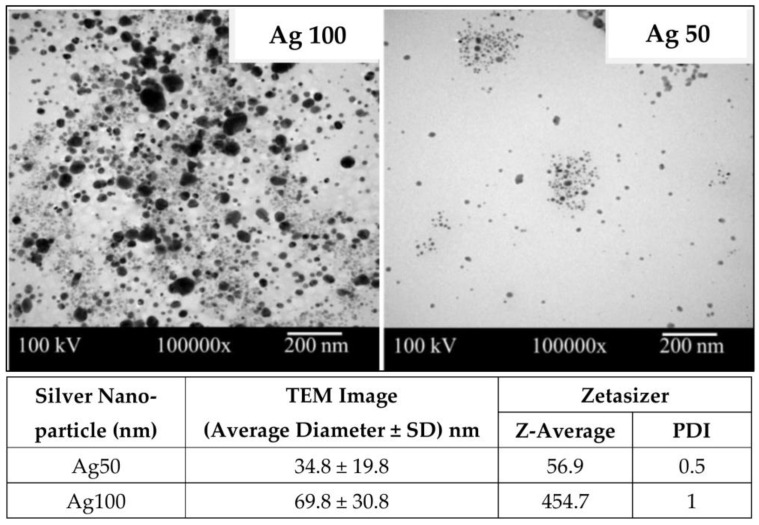
TEM images of silver nanoparticles (AgNPs), Ag100 and Ag50. The average diameters of AgNPs from TEM images were measured by using image J program. The average sizes of AgNPs in DI water were analyzed by using Zetasizer (Malvern Zetasizer ZSP, Worcestershire, UK).

NRL with 60% of dry rubber content (phr) was obtained from the Rubber Research Institute, Bangkok, Thailand. Cellulose fibers (CF) from pulps of Eucalyptus (camaldulensis Dehnh.) were kindly supplied by Teppatana Paper Mill Co., Ltd., Bangkok, Thailand. The pulps were soaked in deionized (DI) water and homogenized at 5000 rpm for 5 min. Then, the pretreated pulps in form of CF suspension were dried at 80 °C for 12 h. After that, dried CF with the approximate size of 20–25 µm was obtained. Spherical AgNPs (Figure 1) at the two different average diameters of 34.8 ± 19.8 and 69.8 ± 30.8 nm (simply coded as Ag50 and Ag100, respectively), were provided in form of solution by Prime-Nanotechnology Co., Ltd., Bangkok, Thailand. SCMC (average MW of ~250 kDa and degree of substitution of 0.9), 3-(4,5-Dimethyl-2-thiazolyl)-2,5-diphenyl 2H-tetrazolium bromide (MTT), and other chemical reagents were purchased from Sigma-Aldrich (Bangkok, Thailand) Co., Ltd. Dulbecco’s Modified Eagle Medium (DMEM), Fetal Bovine Serum (FBS), and antibiotic-antimycotic were purchased from Gibco, Inc., Waltham, MA, USA. Bacterial cells were purchased from American Type Culture Collection (ATCC). The antibacterial activities were evaluated against *Escherichia coli* (*E. coli*; ATCC 25922) and *Staphylococcus aureus* (*S. aureus*; ATCC 25923). Human immortalized keratinocyte cell line (HaCaT) used for cytotoxicity testing were obtained from CLS-Cell Lines Services, Eppelheim, Germany.

### 2.2. Preparation of the Composite Films

SCMC (1 g) was dissolved in 100 mL of DI water prior to dispersing 1.2 g of dried CF by ultrasonic processor (450 W, 42% amplitude, Sonic, Bangkok, Thailand) for 3 min according to the previous research [12]. AgNPs in the form of a solution were added into the mixture of NRL and CF; the solutions of Ag50 and Ag100 contain the solid content of Ag nanoparticles at 2.20 and 2.89% *w*/*w*, respectively. AgNPs at the different weight ratios were added into the mixture under stirring at 500 rpm. NRL (1.3 g) was gradually added into the cold ternary mixture, previously kept refrigerated at 4 ± 1 °C for an hour, while stirring at 500 rpm. The mixture was degassed in an ultrasonic bath for 10 min before pouring into a polystyrene petri dish for drying at 40 °C, 48 h. The composite films were named based on the particle sizes and concentrations of AgNPs as listed in Table 1.

### 2.3. Physical and Chemical Characterization

The opacity of the composite films was measured by using the UV-Vis spectrophotometer (UV-2450, Shimadzu, Kyoto, Japan). A film sample was set perpendicular to the light source at the wavelengths of 200–800 nm. The opacity was then measured by the absorbance at wavelength of 600 nm/unit thickness of the film.

Surface and cross-sectional morphologies of the composite films were studied by Field Emission Scanning Electron Microscopy (FE-SEM, JEOL JSM-7610F, Oxford, UK). The sample was frozen in liquid nitrogen, cryogenically fractured, and then dried under vacuum condition for the cross-sectional morphology. The samples were then coated with a thin layer of gold and imaged at 5 kV.

Fourier transform infrared spectra were obtained by using Attenuated Total Reflectance Fourier Transform Infrared spectrometer (ATR-FTIR, Perkin Elmer, Beaconsfield, UK) within the wavelength between 4000 and 500 cm^−1^, a resolution of 4 cm^−1^ for 30 scans.

Crystallinity was characterized by X-ray diffractometer (XRD, Bruker AXS Model D8 Discover, Bremen, Germany) equipped with VÅNTEC-1 detector at 40 kV and 40 mA. The relative intensity was recorded in a step of 0.02°. The XRD data were analyzed by Rietveld analysis [12]. The crystallinity (%) was obtained by comparing the area under crystalline peaks and the total area.

Young’s modulus, tensile strength, and elongation at break were measured by the Universal Testing Machine (Instron 5582, High Wycombe, UK). The test was performed by following ASTM D882. The samples were prepared in form of a rectangular sheet of 1 × 10 cm^2^. The test was performed at ~25 °C with applied load of 5 kN and a crosshead speed of 0.1 mm/s.

The glass transition temperature (T_g_) was detected from Differential Scanning Calorimeter (DSC, NETZSCH DSC 204 F3, Waldkraiburg, Germany). The biocomposite films were heated from −100 to 300 °C under N_2_ atmosphere at the heating rate of 10 °C/min. Decomposition temperature (T_d_) and residual weight were detected from Thermogravimetric Analyzer (TGA, NETZSCH TG 209 F3, Germany). The film sample with weight of 9–10 mg was heated at 35–600 °C under N_2_ atmosphere at the heating rate of 10 °C/min. The decomposition temperature (T_d_) at the different percentages of weight loss was determined from the TGA curves.

Water absorption capacity (*WAC*) was evaluated by immersing the dry film sample (2.5 × 2.5 cm^2^; recorded the initial weight as *W*) in 100 mL DI water. After a week, excess water of the hydrated sample was blotted using Kimwipes^®^ paper and the weight of the wet sample was recorded as *W_w_*. Water absorption capacity was calculated by:

$$WAC\;\left(\%\right)=\;\frac{W_{w}-\;W}{W}\;\;\times \;100$$

Toluene uptake was determined by immersing the dry film sample (2.5 × 2.5 cm^2^) in 10 mL toluene solution for 8 h. During the immersion, the sample was periodically weighed every 2 h and recorded the weight as *W_t_*. The toluene uptake was calculated by:

$$Toluene\;uptake\;\left(\%\right)=\;\frac{W_{t\;\;}-\;W\;}{W}\;\times \;100$$

Degradability in soil of biocomposite films was tested for 2 weeks. The dry film sample (2.5 × 2.5 cm^2^) was measured for the initial dry weight (*W*_0_) before burying in 100 g natural soil in a 5.5 × 4.5 × 5.0 cm^3^ box, at a depth 10 cm from the soil surface, under ambient condition (35 ± 3 °C, 75% humidity). After 2 weeks, the samples were removed from the soil, washed with DI water, dried at 40 °C for 12 h, and measured for the leftover weight (*W_l_*). The weight loss of the sample was calculated by:

$$Weight\;loss\;\left(\%\right)=\;\frac{W_{o\;\;}-\;W_{l}\;}{W_{0}}\;\times \;100$$

### 2.4. Biological Characterization

The antimicrobial properties of composite films against *E. coli* and *S. aureus* were assessed based on the modified method of AATCC100 [27]. Briefly, the stock cell suspension of *E. coli* and *S. aureus* were prepared at 37 °C and for 16–20 h incubation. The composite film was cut in the square swatch (5 × 5 cm^2^) and inoculated with 1 mL of the bacterial cell suspension at the initial cell density of 2.5–10 × 10^6^ CFU/mL. After incubation at 37 °C for 24 h, 10 mL of phosphate buffered saline pH 7.4 (PBS) was added to the swatch and shake vigorously at 200 rpm for 1 min. The cells in PBS were cultured on the agar plate and incubated at 37 °C for 24 h before counting the cell colonies.

The cytotoxicity of the composite films was tested against HaCaT cells. The sterilized dried film having a diameter of 16 mm was aseptically transferred to a 24-well tissue polystyrene plate. The sterile metallic rings were added to each well to sink the samples before cell seeding. The cells at a density of 1 × 10^5^ cells/mL in DMEM media containing 1% *v*/*v* antibiotic and 10% *v*/*v* FBS were seeded to each well and incubated at 37 °C in a humidified atmosphere composed of 5% CO_2_ for 24 h. The cell viability was determined by using MTT assay at a wavelength of 570 nm. The cell morphology was observed by FE-SEM (JEOL JSM-7610F, Tokyo, Japan). The cells were fixed using 2.5% *v*/*v* glutaraldehyde solution in 0.1 M PBS for 2 h, and rinsed with PBS and DI water, respectively. The fixed cells were dehydrated by immersion in a series of graded ethanol sequentially and dried in a critical point dryer (Leica EM-CPD300, Jurong East, Singapore). The samples were coated with a thin layer of gold in a sputter coater (Balzer SCD-040, Wetzlar, Germany) before scanning at a magnification of 2000 with an accelerating voltage of 10 kV.

### 2.5. Statistical Analysis

Statistical analysis was performed by using the Meet Minitab Release 1.4 software (Minitab Inc., Cray, NC, USA) based on a two-sample *t*-test. The differences between two groups were considered statistically significant at a *p*-value of 0.05 (*p* < 0.05).

## 3. Results and Discussion

### 3.1. Opacity and Morphology

**Figure 2:**
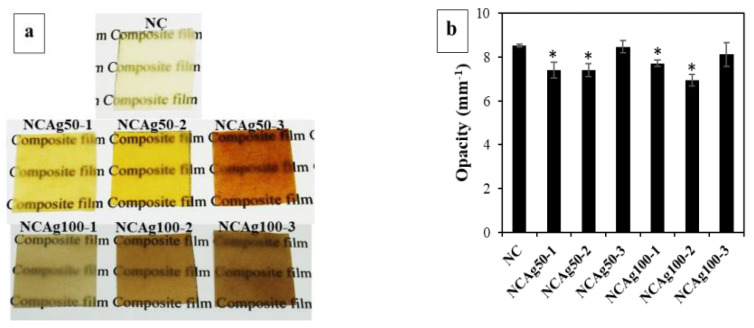
Photographs (**a**) and degree of opacity (**b**) of NC, NCAg50, and NCAg100 films. The symbol * indicates a significant difference (*p* < 0.05, *n* = 5) versus NC.

AgNPs with different amounts (1, 2, and 3 mg or 0.033, 0.067, and 0.1% *w*/*w*, respectively) and particle sizes (ca. 50 and 100 nm) were additionally incorporated into NR, CF, and SCMC ternary composite mixture to obtain the antibacterial film. The effects of the incorporation of AgNPs on the opacity and morphology of the composite films were determined as seen in Figure 2. Figure 2a illustrated color changes of the film from typically translucent with light yellow to dark brown. The color of the films became darker after adding the higher concentration of AgNPs due to surface plasmon resonance property of AgNPs [28]. When the larger Ag nanoparticles comprised in the films (NCAg100), the films appeared darker in color than those of the smaller particle size (NCAg50). This might be due to the boarder and larger particle size distribution of Ag100 (Figure 1). The degrees of opacity of NC and NCAg50-3 were 8.5 mm^−1^ (Figure 2b), while those values of NCAg50-1 and NCAg50-2 dropped to about 7.4 mm^−1^. A similar trend was noticed for the NCAg100 films. This could possibly be influenced by chemical composition, structure, and thickness of the films (Figure S1).

**Figure 3:**
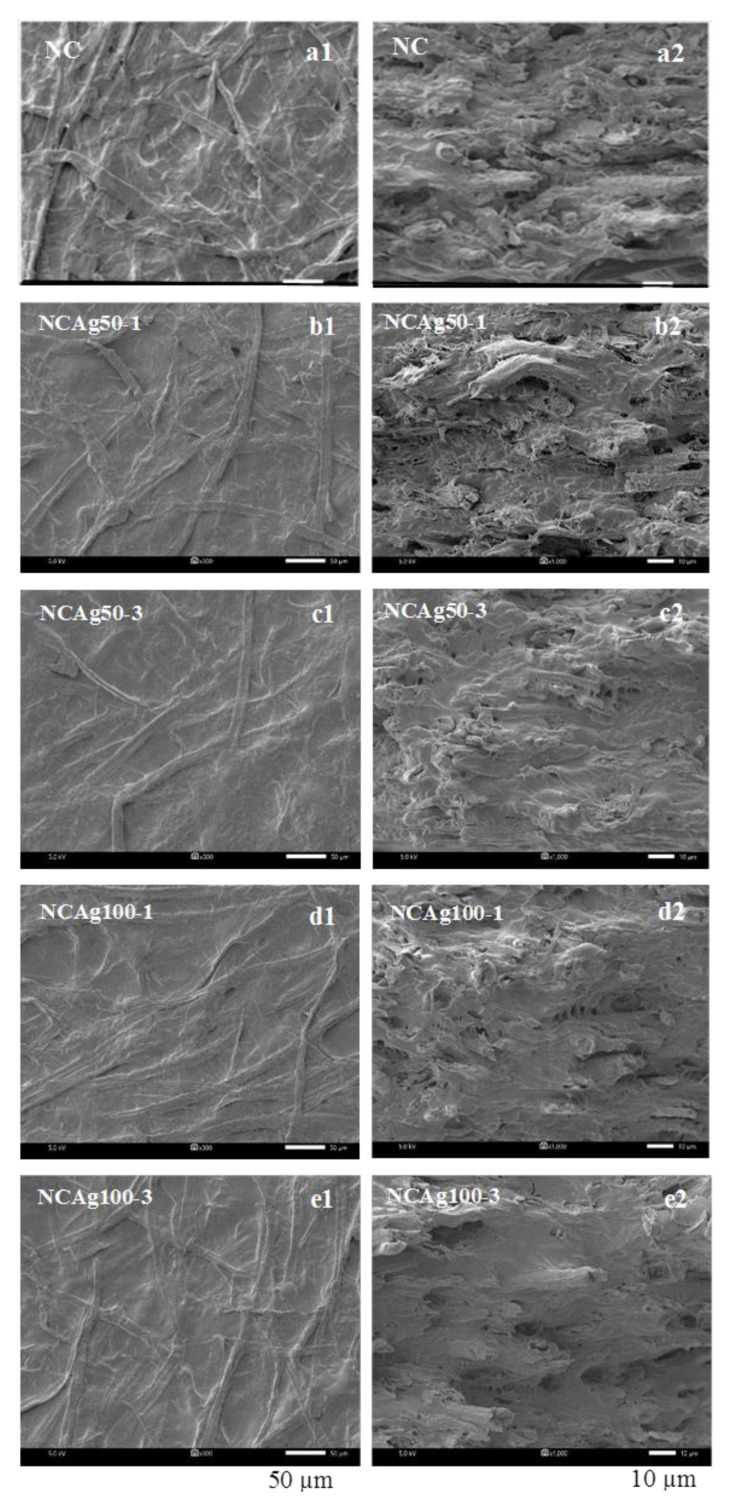
SEM images of surface (a1–e1), (**left**) and cross-section (a2–e2), (**right**) of NC, NCAg50, and NCAg100 films.

Surface morphology was observed by SEM as shown in Figure 3. Cellulose fibers (CFs) were uniformly distributed in the NC film. After blending with AgNPs, the surface morphology of all the NCAg composites became relatively smoother. This might imply better homogeneous interactions among the components of the NCAg composite films. It was noticed that various concentrations and particle sizes of loaded AgNPs under the studied conditions did not apparently affect the surface morphology of the composites. For the cross-sectional morphology, there was no phase separation between the two components (i.e., cellulose and NR) of which polarity and density were different due to the addition of SCMC [12]. There was no significant difference in the cross-sectional morphology of NC films and the NCAg composites.

### 3.2. Chemical Interaction

**Figure 4:**
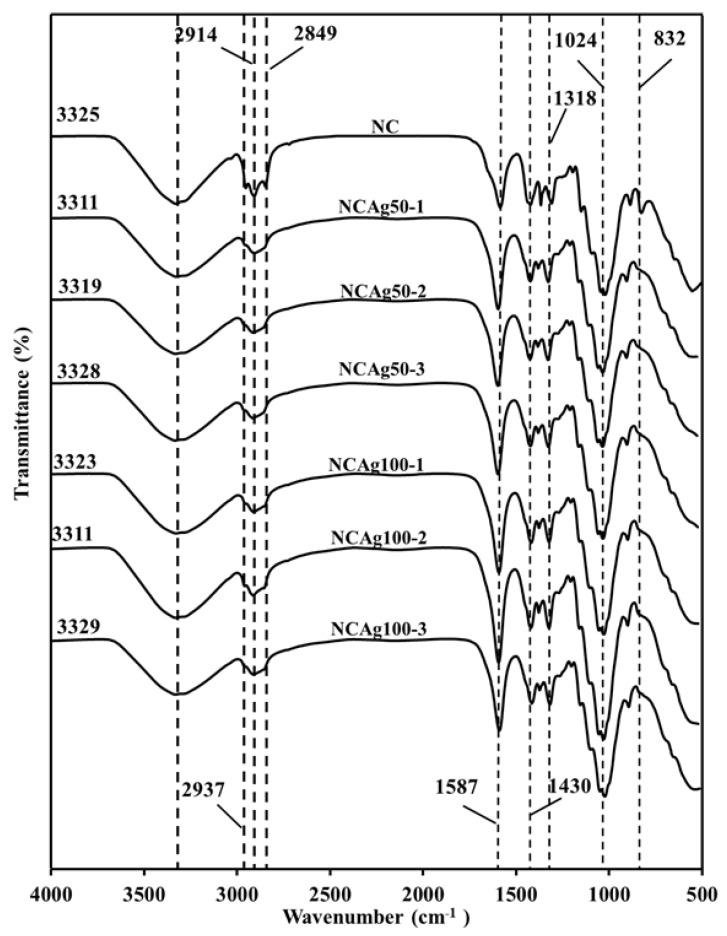
FTIR spectra of NC, NCAg50, and NCAg100 films.

The FTIR spectra for NC, NCAg50, and NCAg100 composite films are shown in Figure 4. All the spectra in the composites showed the characteristic absorption bands of NR, CF, and SCMC. The peaks at about 832, 1024, 1338, and 1430 cm^−1^ were ascribed to =C–H out-of-plane bending, C–C stretching, –CH_3_ bending, and –CH_2_ bending, respectively, which were the characteristic peaks of NR [29,30]. The sharp peaks in the spectral region of 2849, 2914, and 2937 cm^−1^ were ascribed to –CH_2_ symmetric stretching, –CH_2_ asymmetric stretching, and –CH_3_ asymmetric stretching, respectively, possibly due to the phospholipid surrounding on the rubber particles [31]. For cellulose components, i.e., CF and SCMC, characteristic peak stretching vibration of the hydroxyl group was indicated at 3510–3094 cm^−1^. The center of the peak of the NCAg composites slightly shifted from that of NC film located at 3325 cm^−1^ towards the lower wavenumber peaks at 3329–3311. This might be caused by the effect of AgNPs on hydrogen bonding between hydroxyl group of cellulose and amino group of protein in NR. As compared to FTTR spectrum of NC film, there was no major shift of peaks and no increase in peak intensity among the characteristic peaks of cellulose. This may confirm that the chemical interaction between AgNPs and cellulose did not occur. However, with the addition of AgNPs, the peak at 1587 cm^−1^ assigned for protein in NR was relatively shaper and had a minor shift, which possibly indicated some interaction between protein in NR and AgNPs. It was reported that protein could bind to nanoparticles through free amine groups [28].

### 3.3. Crystallinity

**Figure 5:**
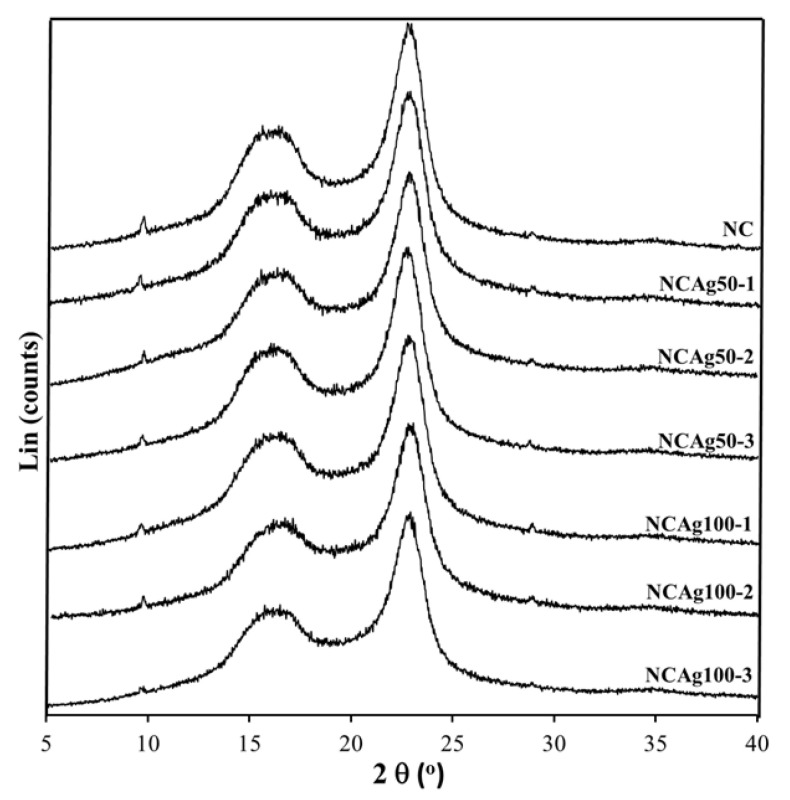
XRD patterns of NC, NCAg50, and NCAg100 films.

From XRD diffractograms of NC and NCAg composite films (Figure 5), crystalline peaks located at about 17° and 23° displayed the typical pattern of cellulose I allomorph having major reflection planes at 110 and 200, respectively [32]. A broad peak centered at 2θ of ~18° indicating an amorphous halo of NR was hardly seen in all the composites, whereas it was clearly shown in the neat NR sample [12]. Because the loading content of AgNPs in the composites was very low (0.033–0.10% *w*/*w*), a crystalline peak at 2θ of ~38° indexed to 111 orientations of AgNPs [28] could not be observed on the XRD patterns of the composites. From Table 2, the highest crystallinity values of 72% belonged to the NC film due to the high weight proportion of crystalline cellulose (40% *w*/*w*) containing in the film sample. The crystallinity values slightly decreased according to small amount of added AgNPs. As compared among NCAg composites, the crystallinity values of NCAg50 and NCAg100 reduced to 64.8–69.1% and 65.6–70.0%, respectively. This observation indicated that AgNPs might interfere with hydrogen bonding interactions between CF, SCMC, and NR in composite films [33].

### 3.4. Mechanical Properties

**Figure 6:**
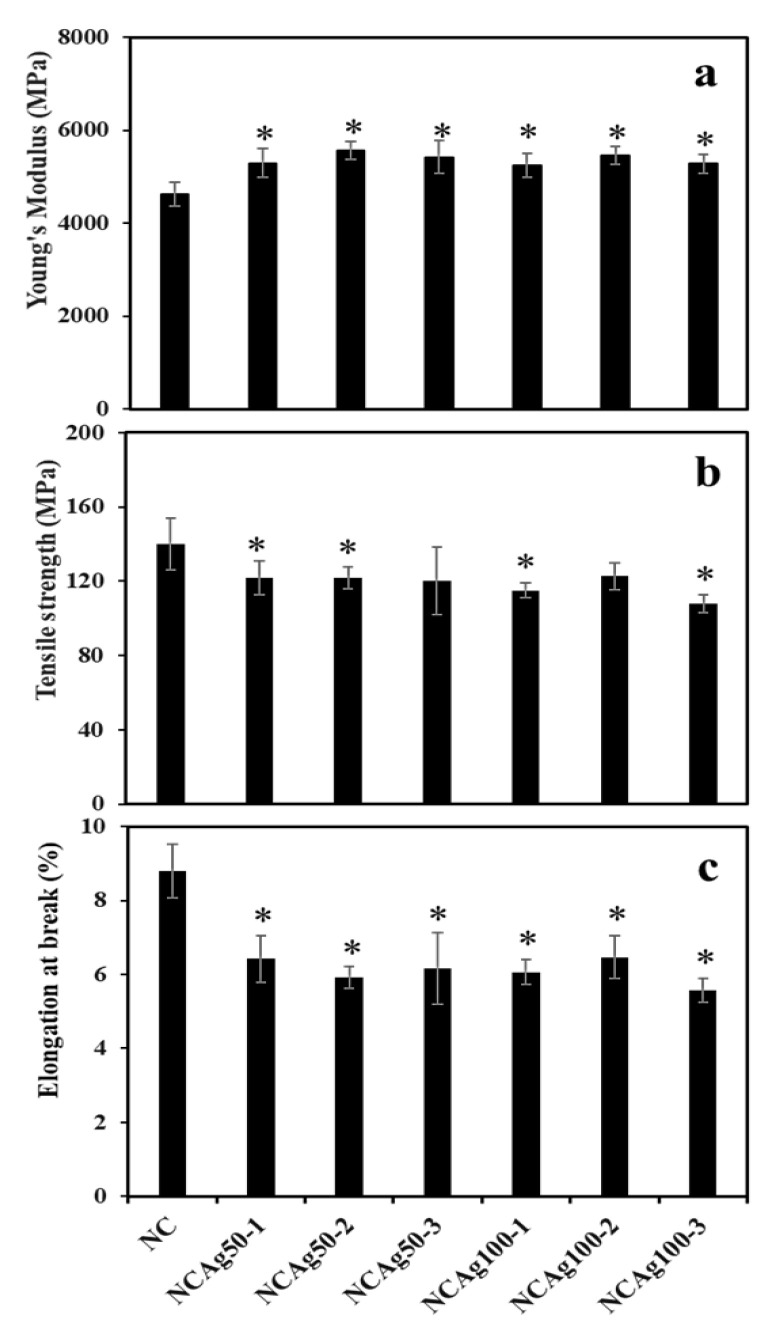
Young’s modulus (**a**), tensile strength (**b**), and elongation at break (**c**) of NC and NCAg composite films. The symbol * indicated a significant difference (*p* < 0.05, *n* = 5) vs. NC film.

The mechanical properties of NC and NCAg composite films were investigated in terms of Young’s modulus, tensile strength, and elongation at break (Figure 6). The Young’s modulus of the NC film was 4625 MPa (Figure 6a). The films became harder with the improved modulus value to approximately 5000 MPa, when adding AgNPs. Particle sizes and concentrations of AgNPs slightly affect the moduli of NCAg films. NCAg50 films possessed relatively higher modulus values as compared with NCAg100 films containing the same concentration of AgNPs. It was noted that the high moduli were obtained from NCAg50-2 and NCAg100-2. In Figure 6b, the tensile strength of NCAg50 and NCAg100 films dropped about 10–20% lower than that of the NC film (140 MPa); the results showed a significant decrease in those of NCAg50-1, NCAg50-2, NCAg100-1, and NCAg100-3. This could imply that AgNPs did not mainly support reinforcement effect of the metallic nanoparticles in organic NR-cellulose matrix. According to FTIR result, no chemical interaction between AgNPs and the matrix was observed. However, AgNPs might affect the hydrogen bond strength of CF, SCMC, and NR in composite films. Consequently, compared to NC films, the tensile strength of NCAg films relatively decreased, in a similar way as being observed in the degrees of crystallinity. However, these two values i.e., Young’s modulus and tensile strength of NCAg composites were much greater than those of the neat NR film (19 and 0.8 MPa, respectively) [12]. In Figure 6c, elongation at break values were in the same trend as tensile strength. The values were about 1.3 times dropped from that of NC film (8% elongation at break). Even though NR had high elasticity and SCMC acted as a plasticizer enabled to improve the elasticity, CF could form a rigid network in the rubber matrix resulting in an obstructed mobility of NR polymer chain [12]. The addition of Ag50 and Ag100 at the studied concentration also showed a significant reduction of the elongation at break of NCAg50 and NCAg100 films. However, the mechanical properties of NCAg biocomposite films are excellent and relatively much higher, especially for tensile strength and modulus as compared to those of many other biocomposite films previously reported [18,19].

### 3.5. Thermal Property

**Figure 7:**
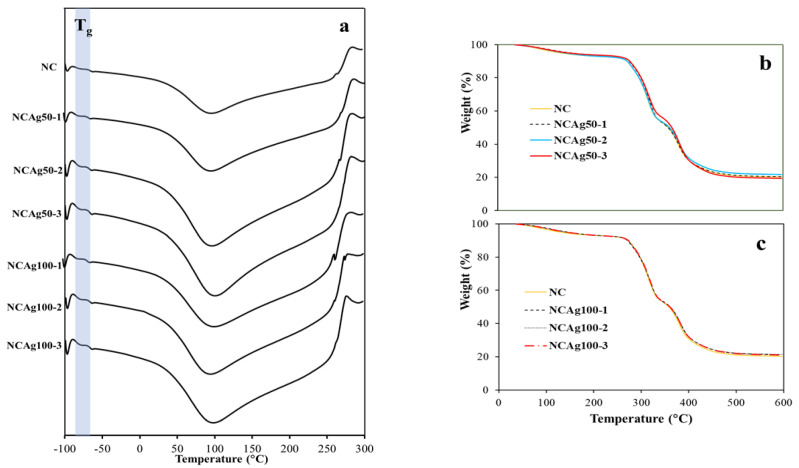
DSC thermograms of NCAg50 and NCAg100 films (**a**); TGA curves of NCAg50 films (**b**) and NCAg100 films (**c**) as compared to NC film.

The glass transition (T_g_) obtained from DSC thermograms (Figure 7a) of NC and the composite films are tabulated in Table 2. The Tg of NC film was −64.3 °C, which was about 2 °C higher than NCAg films. This was possibly because AgNPs interfered hydrogen bonding arrangement in the composite, resulting in lower crystallinity and consequently lower T_g_. According to the similar T_g_ values among the composites, the concentration and particle size of AgNPs applied in this study did not significantly affect T_g_ of these composite films. From the thermograms, the endothermic peaks indicating from the sharp decrease in temperature at about 100 °C showed the desorption of water molecules in the NC and the composites. The exothermic degradation peaks were observed at ~285 °C.

Thermal properties of NC and NCAg composite films were studied by TGA and DSC. The TGA curves of NCAg50 and NCAg100 films are shown in Figure 7b,c, respectively. At the initial temperature ranging from 40 to 270 °C, the weight losses of all the samples were caused by dehydration of moisture. From the overlapped curves, the thermal stability of all the samples was quite similar, possibly as a result of only low concentration of AgNPs incorporated in the composite. The TGA curves of all the samples demonstrated the two-step thermal decomposition. For NC film, the first and the second steps decomposition temperature was ~315 and 379 °C, respectively (Table 2). Compared to NC film, NCAg composite films showed higher decomposition temperatures, but the values were rather similar among them regardless of concentrations and sizes of AgNPs. The first and the second decomposition of the composites occurred at the temperature of around 316–319 and 381–383 °C, respectively. The total weight loss at 450 °C for all the samples was ~80%. The range of thermal stability for the NCAg composite films was 0 to 260 °C; therefore, the composite films could be sterilized under the normal steam sterilization condition of medical and food packaging products (121 °C).

### 3.6. Degradation in Soil

**Figure 8:**
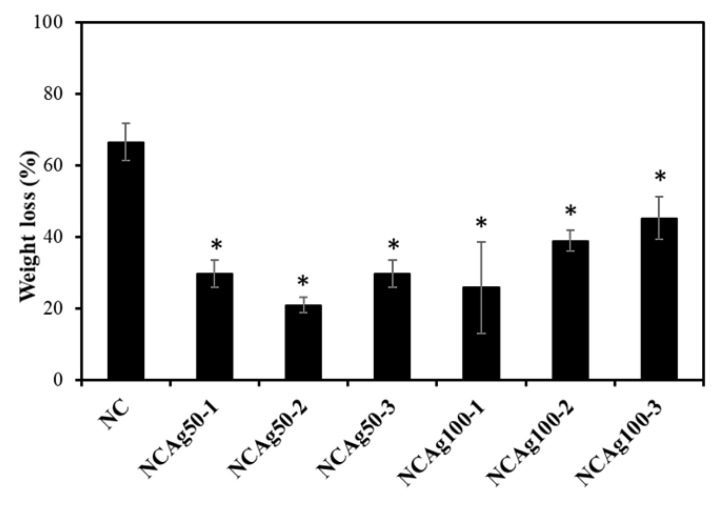
Biodegradation in soil of NC, NCAg50, and NCAg100 films after 2 weeks. The symbol * indicates a significant difference (*p* < 0.05, *n* = 3) vs. NR films.

Biodegradability is an important property of eco-friendly materials. Since the use of carbon source from NR of the microorganisms is quite slow, the NR-based material degraded slowly in nature. Addition of cellulose induced more rapid biodegradability [12]. In Figure 8, the degradation in soil of NC sample was 65% in 2 weeks without additionally inoculated with bacterial cells. The degradation of NCAg composites showed a significant reduction in weight loss in soil. After 2 weeks, the degradation of NCAg composites in soil was about 20–40% of the initial weight (about 31–62% of that of NC films). The lower biodegradation rate should be caused by the antibacterial effect of AgNPs embedded in the composite films.

### 3.7. Toluene Uptake and Water Absorption Capacity

The uptake of toluene, an excellent solvent for nonpolar NR products, was characterized as a function of time. NR film dissolved almost completely in toluene within 4 h, whereas the swelling behaviors of NC, NCAg50, and NCAg100 films were slightly noticed. NC sample gradually swelled in toluene and reached 20% of toluene uptake after the immersion for 6–8 h due to hindered toluene diffusion of cellulose (Table 3). The resistance of toluene uptake was enhanced by the integration of AgNPs into the composites. After 2 h, toluene uptake of NCAg films was only ~7–12% and remained this value until 8 h. It was shown that the films integrated with higher concentration of AgNPs (NCAg50-3 and NCAg100-3) could be more resistant to toluene, which is a non-polar solvent. However, no significant difference in the toluene uptake of NCAg50 films and NCAg100 films was observed.

The water absorption capacity of the NCAg composite films was around 200%, which tended to decrease comparing with the NC film (~280%) as seen in Table 2, possibly as the result of the interactions of AgNPs with other components in the films. It was suggested that negative carboxylate charges (–COO−) of SCMC could neutralize the positive charges of the unchelated silver ions (Ag^+^) [34]. In this study, the release of AgNPs from the films into PBS pH 7.4 using modified Franz diffusion cells was analyzed by ICP-OES measurement (Table S1). The release of AgNPs from NCAg50-1, NCAg50-2, NCAg100-1, and NCAg100-2 was very low (<5.0 µg/L), thus it could not be detected by ICP-OES measurement. However, the release of AgNPs from NCAg50-3 and NCAg100-3 could be detected. After 48 h, the cumulative releases of AgNPs of NCAg50-3 and NCAg100-3 were 16.4 and 6.2 µg/L (or 0.20 and 0.07%), respectively. Therefore, at the highest AgNPs loading (0.1% *w*/*w*), AgNPs could slightly release from the NCAg films and the rate of the release of the smaller nanoparticles (Ag50) from the films was higher than that of the larger one (Ag100).

### 3.8. Antibacterial Activity

**Figure 9:**
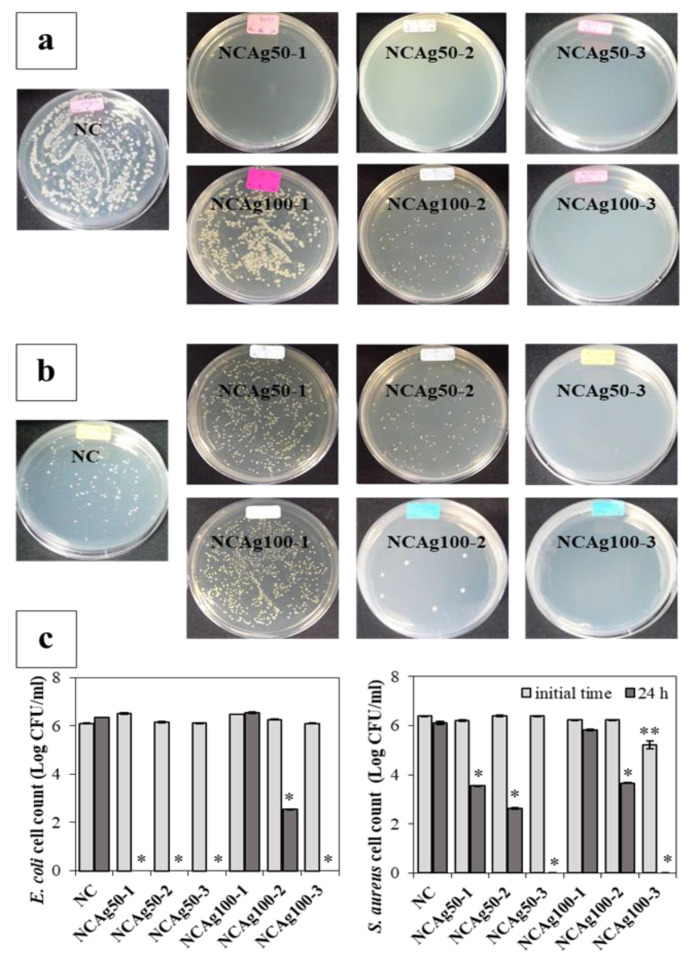
Antibacterial activity of NC and NCAg films against *E. coli* (**a**) and *S. aureus* (**b**) investigated by the plate count method, and quantitative colony counting (**c**) at 0 h (■) and 24 h (■). The symbols ** and * indicate a significant difference (*p* > 0.05, *n* = 3) vs. NC at 0 h and 24 h, respectively.

NCAg composites exhibited a strong antimicrobial activity against *E. coli* and *S. aureus*, used as models of Gram-negative and Gram-positive bacteria, after incubation for 24 h (Figure 9a,b, respectively). The number of colonies on the plates was counted as shown in Figure 9c. Comparing with NC film, all NCAg50 films strongly exhibited antibacterial property against *E. coli*, of which the colony could not be visible on the agar plate and counted (Figure 9a). The antibacterial activity was relatively lower for the films loading with the larger Ag ions (NCAg100). No antibacterial activity against *E. coli* was observed on NCAg100-1. However, after incubation for 24 h on NCAg100-2, *E. coli* colonies significantly decreased (from ~10^6^ CFU/mL to less than 10^3^ CFU/mL) and were more strongly inhibited on NCAg100-3 (from ~10^6^ CFU/mL to 0 CFU/mL). In Figure 9b, *S. aureus* was also sensitive to NCAg films. The reduction of the Gram-positive bacteria also related to the size of AgNP and the concentration. NCAg100-1 and NCAg100-2 reduced colony numbers of *S. aureus* from 10^6^ CFU/mL to less than 10^6^ and 10^4^ CFU/mL, respectively, whereas *S. aureus* were more susceptible to NCAg50-1 and NCAg50-2 (colony numbers were reduced to less than 10^4^ and 10^3^ CFU/mL, respectively). A very strong inhibition effect was observed after 24 h of the incubation on the NCAg films containing the highest AgNPs content at 0.1% *w*/*w* (NCAg50-3 and NCAg100-3), where no colonies could be detected on the agar plates. The mechanism of antimicrobial activity involved the interaction of Ag^+^ with biological macromolecules e.g., protein. The monovalent Ag^+^ replaced hydrogen ions (H^+^) of thiol groups of bacterial cell membrane resulting in inactivating the cell protein, decreasing membrane permeability, and eventually causing cellular death [35]. In this study, it was shown that the smaller Ag nanoparticles (Ag50) exhibited higher antibacterial activities than the larger one (Ag100).

### 3.9. Cytotoxicity

**Figure 10:**
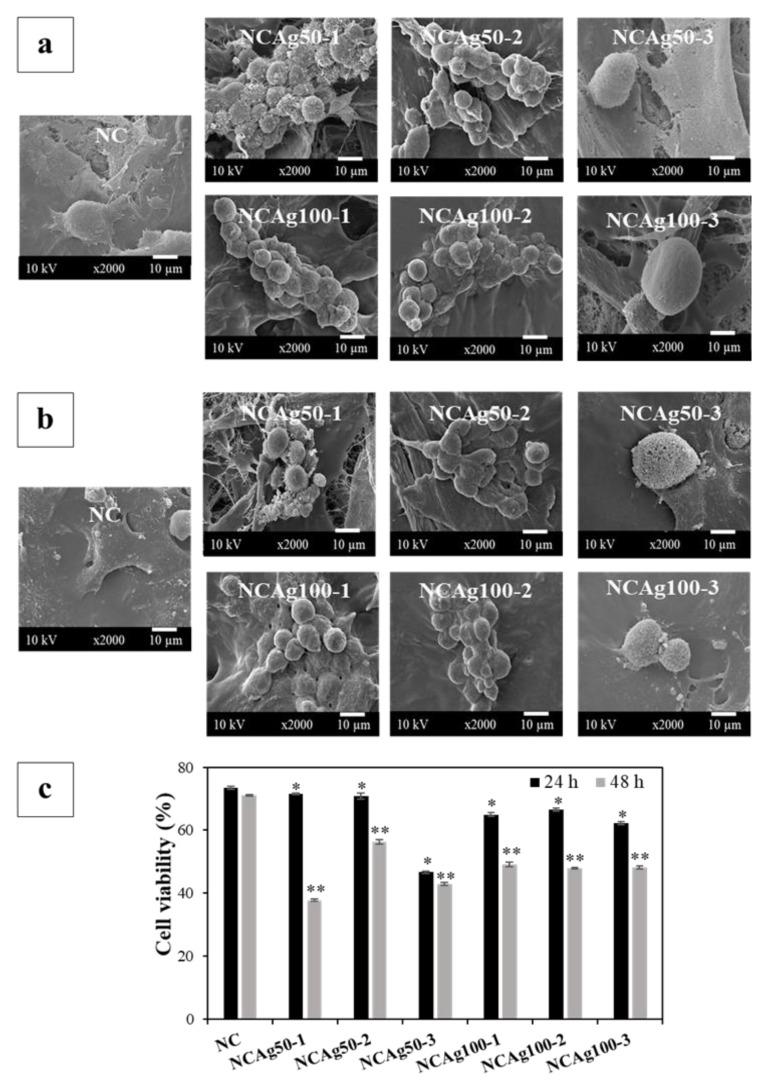
Morphology of HaCaT cells cultured on NC and NCAg composite films for 24 h (**a**) and 48 h (**b**). Cell viability was quantified (**c**) at 24 h (■) and 48 h (■). The viability was normalized by the viability of cells cultured on tissue culture plate, which was expressed as 100% viability. The symbols * and ** indicate a significant difference (*p* > 0.05, *n* = 3) vs. NC at 24 and 48 h, respectively.

Morphology and viability of the human keratinocyte cell line, HaCaT, cultured on NC and NCAg composite films for 24 and 48 h, were assessed. In Figure 10a,b, normal morphology of HaCat cells was observed on NC films at 24 and 48 h. The cells spread well with lamellipodia and filopodia and had contact with surrounding cells indicating the healthy cells. On the other hand, on NCAg50-1 film at 24 h, the round cells with filopodial protrusion were observed (Figure 10a), and cell viability was about 72% (Figure 10c). Due to higher loading of AgNPs in NCAg50-2 and NCAg50-3, viabilities of the cells dropped to 70 and 47%, respectively. However, viabilities of cells cultured on NCAg100 were ~60%, regardless of AgNPs content (0.033–0.10% *w*/*w*). Therefore, the larger AgNPs might be slightly less toxic to HaCaT cells possibly involved in lower cell membrane permeability causing less cell damage [36]. At 48 h (Figure 10b), round cells were observed on all the samples and cell viability reduced as compared to those cultured for 24 h. It was previously reported that AgNPs could weaken cell adhesion on films [37]. However, besides NCAg50-1, about 50% of cells were viable on the NCAg composite films suggesting the moderate cytotoxicity.

## 4. Conclusions

Composites of cellulosic natural rubber and silver nanoparticle were successfully prepared by simple blending method using carboxymethyl cellulose as a dispersing agent via a latex aqueous microdispersion process. Tensile strength, crystallinity, and water absorption capacity of the composite slightly dropped after adding silver nanoparticles, but remained excellent values resulted from the cellulose microfiber reinforcement. The composites exhibited biodegradability and heat resistance. Toluene resistance was substantially improved by the integration of silver nanoparticles with the size of ~50 to 100 nm. It is demonstrated that effective antibacterial properties of biocomposite films of NR and CF could be developed by applying very low content of silver nanoparticles at 0.033–0.1% (*w*/*w*). NCAg composites exhibited a strong antimicrobial activity against *E. coli* and *S. aureus* and the films loading the smaller Ag nanoparticles (NCAg50) presented more enhanced antibacterial properties as compared to those with the larger Ag nanoparticles (NCAg100). In addition, NCAg biocomposite films showed low cytotoxic effects on human skin HaCaT keratinocyte cells. From the in vitro cytotoxicity testing, after being cultured on NCAg composite films for 24 and 48 h, HaCaT keratinocyte cells were good and moderately viable on the films, respectively. According to the improved properties, the composite films of NCAg have good potential applications in antimicrobial packing material and antimicrobial wound care products.

## Figures and Tables

**Table 1:** Contents of NR, CF, SCMC, and AgNPs in the dried composite films.

Samples	NR (g)	CF (g)	SCMC (g)	Ag50 (mg)	Ag100 (mg)
NC	0.8	1.2	1.0	-	-
NCAg50-1	0.8	1.2	1.0	1.0	-
NCAg50-2	0.8	1.2	1.0	2.0	-
NCAg50-3	0.8	1.2	1.0	3.0	
NCAg100-1	0.8	1.2	1.0	-	1.0
NCAg100-2	0.8	1.2	1.0	-	2.0
NCAg100-3	0.8	1.2	1.0	-	3.0

**Table 2:** Degree of crystallinity, decomposition temperature (T_d_), glass transition temperature (T_g_), and water absorption capacity (WAC) of NC, NCAg50, and NCAg100 films.

Samples	AgNPs (% *w*/*w*)	Crystallinity (%)	T_d_ (°C)		T_g_ (°C)	WAC (%) (After 7 Days)
			1st Weight Loss	2nd Weight Loss		
NC	0.000	72.0	314.8	378.9	−64.3	279.1 ± 27.1
NCAg50-1	0.033	64.8	318.7	380.8	−66.6	188.8 ± 29.2
NCAg50-2	0.066	64.9	316.9	382.0	−67.0	239.9 ± 48.6
NCAg50-3	0.100	69.1	317.6	381.8	−66.4	180.6 ± 37.3
NCAg100-1	0.033	65.6	316.1	382.3	−66.2	215.3 ± 31.0
NCAg100-2	0.066	65.7	317.0	381.9	−66.7	259.0 ± 47.0
NCAg100-3	0.100	69.9	316.7	382.6	−66.1	218.8 ± 82.6

Note: The degree of crystallinity was calculated by TOPAS Rietveld analysis software on X-ray diffractometer; Td and Tg were detected from thermogravimetric analysis (TGA) and differential scanning calorimetry (DSC) thermograms, respectively.

**Table 3:** Percentage of toluene uptake of NC, NCAg50, and NCAg100 after the immersion for 2, 4, 6, and 8 h in toluene. The data were the average values determined from three specimens (*n* = 3).

Samples	AgNPs (% *w*/*w*)	Time (h)			
		2	4	6	8
NC	0.000	3.6 ± 1.7	13.6 ± 3.9	19.2 ± 3.1	20.0± 4.0
NCAg50-1	0.033	11.0 ± 3.1	10.2 ± 4.0	11.7 ±1.1	8.4 ± 1.7
NCAg50-2	0.066	9.3 ± 2.0	8.7 ± 1.4	8.9 ± 1.7	8.7 ± 2.5
NCAg50-3	0.100	5.0 ± 1.8	7.2 ± 2.1	7.3 ± 2.7	6.7 ± 1.0
NCAg100-1	0.033	8.3 ± 2.6	7.6 ± 3.8	9.6 ± 0.7	9.6 ± 2.3
NCAg100-2	0.066	8.4 ± 3.9	10.8 ± 5.3	8.6 ± 0.7	7.6 ± 1.5
NCAg100-3	0.100	9.6 ± 2.9	9.7 ± 2.4	7.9 ± 1.3	7.7 ± 3.3

## Supplementary Materials

The following supporting information can be downloaded at: https://www.mdpi.com/article/10.3390/nano13030521/s1, Figure S1: Thickness values of NC, NCAg50, and NCAg100 films; Table S1: Releasing of silver species from NCAg composite film.
