# Supplementary material for: Multifunctional Cellulosic Natural Rubber and Silver Nanoparticle Films with Superior Chemical Resistance and Antibacterial Properties
Source: Nanomaterials (Basel). 2023 Jan 28;13(3):521. doi: 10.3390/nano13030521 (PMC9921950; doi:10.3390/nano13030521)
Supplement: Supplementary file 1 [file nanomaterials-13-00521-s001.zip › nanomaterials-2155099-supplementary.pdf]

Supplementary data

Multifunctional cellulosic natural rubber and silver nanoparticle films with enhanced chemical resistance and antibacterial properties

Goragot Supanakorn[1], Siriporn Taokaew[2] and Muenduen Phisalaphong [1,*]

[1]Department of Chemical Engineering, Faculty of Engineering, Chulalongkorn University, Bangkok 10330, Thailand

[2]Department of Materials Science and Technology, School of Engineering, Nagaoka University of Technology, Nagaoka, Niigata 940-2188, Japan

*Correspondence: muenduen.p@chula.ac.th; Tel.: +662-218-6875

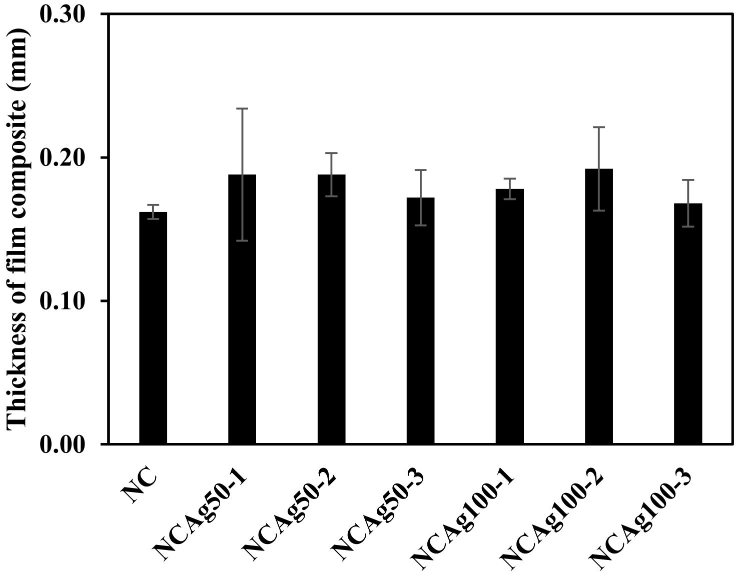

Fig. S1 Thickness values of NC, NCAg50, and NCAg100 films

Table S1. Releasing of silver species* from NCAg composite film.

**Sample**	**Silver species (µg/l)**				
	30 min	6 h	12 h	24 h	48 h
**NCAg100-1**	< 5.0	< 5.0	< 5.0	< 5.0	< 5.0
**NCAg100-2**	< 5.0	< 5.0	< 5.0	< 5.0	< 5.0
**NCAg100-3**	5.0	6.2	5.8	5.8	6.2
**NCAg50-1**	< 5.0	< 5.0	< 5.0	< 5.0	< 5.0
**NCAg50-2**	< 5.0	< 5.0	< 5.0	< 5.0	< 5.0
**NCAg50-3**	5.6	7.4	13.6	18.4	16.4

* The release characteristic of silver species from composite film into phosphate buffered saline (PBS) pH 7.4 was investigated using modified Franz diffusion cells. Rehydrated composite film specimen (32 mm in diameter) was placed on a receptor compartment of the diffusion cell covered with a glass lid. The opening of the donor cell was sealed with a plastic film. The receptor chamber was fully filled with 11.5 mL of PBS. The diffusion cells having water jacketed was maintained at 37 ◦C throughout the experiment. The receptor fluid was kept at 37 ◦C with constant stirring using a Teflon-coated magnetic stir bead. During the release testing (0-48 h), 5 mL sample was withdrawn from the receptor every 6 h through a sampling port and an equal amount of the fresh buffer was immediately refilled. The silver species were detected by using Inductively Couple Plasma Optical Emission Spectrometer (ICP-OES, PQ 9000 elite, Germany).
